# Peripheral Nerve Stimulation in Painful Conditions of the Upper Extremity—An Overview

**DOI:** 10.3390/biomedicines10112776

**Published:** 2022-11-01

**Authors:** Vincent Yaccarino, Max Y. Jin, Alaa Abd-Elsayed, Jacob M. Kraemer, Nalini Sehgal

**Affiliations:** 1 Department of Orthopedics & Rehabilitation, University of Wisconsin Hospitals and Clinics, Madison, WI 53705, USA; 2 Department of Anesthesiology, University of Wisconsin, Madison, WI 53792, USA; 3 SSM Health, Madison, WI 53715, USA

**Keywords:** peripheral nerve stimulation, neuropathic pain, complications

## Abstract

Our objective is to present a brief history of the evolution of peripheral nerve stimulation, the current understanding of peripheral nerve stimulation mechanisms in chronic pain, peripheral nerve stimulation applications in upper extremity chronic pain conditions, and complications of peripheral nerve stimulation. The evolution of peripheral nerve stimulation from the early ages to the current status has been facilitated by discoveries in neurobehavioral mechanisms of pain, advances in technology and percutaneous lead development, and the availability of high-quality portable ultrasound units. Peripheral nerve stimulation application in managing upper extremity pain of amputated limbs, post-stroke shoulder pain, complex regional pain syndrome (CRPS), and median, ulnar, and radial neuropathies are discussed. Finally, we describe complications of peripheral nerve stimulation. The availability of ultrasound-guided peripheral nerve stimulation techniques and superior peripheral nerve stimulation technology have opened up new and minimally invasive treatment options for chronic intractable neuropathic pain of the upper extremity. Additionally, the ability to place peripheral nerve stimulation leads percutaneously without open peripheral nerve surgery expands the pool of implanting physicians, while simultaneously decreasing the risks and complications that are associated with open surgery.

## 1. History of Neuromodulation and Peripheral Nerve Stimulation

Neuromodulation in the treatment of pain dates back thousands of years. The earliest account of electrical stimulation to treat pain was in 15AD when a Roman physician, Scribonius Largus used torpedo fish, a type of electric ray fish, to treat a variety of pain conditions after observing the reduction in gout pain in an individual from accidental contact with a torpedo fish. In 1774, Benjamin Franklin investigated muscle contraction by stimulating with electrical shocks [1]. The widespread therapeutic application of electrical stimulation was delayed until 1848 when Du Bois-Raymond invented the electrical generator [2]. Around this time “medical quackery” was under increased scrutiny and the Flexner Report excluded electrotherapy from clinical practice in 1910 [3].

In 1965, the gate control theory was described by Melzack and Wall and stimulated a resurgence of interest in electrical stimulation to treat chronic pain [4]. In the following decade, various devices, implantation techniques, and applications were described. In 1977, the FDA sponsored a symposium on neuromodulation, where an expert consensus concluded neuromodulation to be safe and effective in the treatment of pain [1].

Throughout the 1980s, the development of commercially available devices such as paddle multi-contact electrodes, percutaneous cylindrical leads, and implantable pulse generator units largely replaced the previously popular cuff-like devices and radiofrequency-coupled devices [5]. However, there were several challenges that limited the use of peripheral nerve stimulation including off-label use, tunneling to the implantable pulse generator, the inability to selectively innervate the sensory components of the target nerve, prolonged operating times, and complications such as lead tethering, lead migration, and wound infection [4]. In addition, long-term success rates were poor [6].

In the late 1990s, interest in peripheral nerve stimulation research resurged with the advent of percutaneous lead placement. Weiner and Reed in 1999 first demonstrated the feasibility of the technique in the treatment of occipital neuralgia [7]. This sparked interest in peripheral nerve stimulation trials for a variety of chronic pain conditions including trigeminal neuralgia, chronic back pain, and complex regional pain syndrome (CRPS), amongst others. Historically, due to the market dominance of spinal cord stimulators, research into peripheral nerve stimulation was largely limited to the adaptation of spinal cord stimulator devices for use in the periphery. This presented a problem as the forces that are applied to the stimulator and leads in the periphery are substantially greater than in the epidural space [8]. This is an exciting time with a multitude of medical and surgical subspecialties interested in chronic pain treatments, advancements in ultrasound technology and expertise in lead placement, and continued advances in peripheral nerve stimulation devices. This increased interest has led to the publication of a previous review that was focused on peripheral nerve stimulation for upper extremity conditions [9]. As compared to this previous review, our review discusses additional studies and highlights important clinical trials in progress to provide a more comprehensive overview of the topic.

## 2. Proposed Mechanism of Action for Peripheral Nerve Stimulation

Similar to traditional spinal cord stimulation (SCS), the original thought was that peripheral nerve stimulation devices also worked via the gate control theory. As alluded to above, this would involve sending electric neurostimulation signals to the surrounding non-nociceptive nerve fibers including A-alpha and A-beta fibers, and inhibiting the transmission of nociceptive signals by the A-delta and C fibers to the brain. However, more recently researchers have found that the gate control theory alone does not explain the mechanism of action of peripheral nerve stimulators [10]. In addition to the gating theory of traditional neurostimulators, another proposed mechanism of peripheral nerve stimulation is the effect on biochemical mediators of pain, and alteration in the ion channels that are involved in the transmission of chronic pain signals in the peripheral nervous system and central nervous system. The local tissue inflammation factors as shown in chronic pain conditions can lead to central changes in pain perception. Peripheral nerve stimulation systems may be directly inhibiting local neurotransmitters and endorphins that are linked to chronic pain and may work in conjunction with the gating theory [11,12]. It is suggested that peripheral nerve stimulation systems can have central neuroplastic effects to change pain states and this may explain the continued pain relief that is experienced by patients following peripheral nerve stimulation lead removal. Novel waveforms that include high-frequency peripheral nerve stimulation and burst peripheral nerve stimulation have similar proposed mechanisms of action. It has been proposed that high-frequency peripheral nerve stimulation works through the gate control theory and an alteration in ion channels resulting in a hyperpolarized resting potential [13]. Burst peripheral nerve stimulation has also been proposed to be effective through the gate control theory in addition to the endogenous opioid pathway [14].

In addition to the biological implications of the gate control theory, there are also social and psychological aspects in the modulation of pain. The social aspect is embodied by the biopsychosocial model of pain that discusses how individual experience has an impact on pain. It is also believed that emotions and other aspects of cognition play a role in the perception of pain. This psychological aspect drove the development of the Neuromatrix model of pain (NMP). The NMP builds on the gate control theory and draws on concepts such as the body self-complex [15]. In a recent article by Ramezannezhad et al., two studies were presented that reported correlations between psychological factors and neuromodulation intervention outcomes [16]. One study found that SCS outcomes could be predicted by sleep disturbances [17]. The other study reported that after SCS intervention, psychological scores improved along with pain scores [18]. As previously mentioned, peripheral nerve stimulation follows similar mechanisms as SCS. Therefore, it is reasonable to believe similar results can be achieved for patients that are undergoing peripheral nerve stimulation intervention. These findings have led to an ongoing clinical trial (ClinicalTrials.gov Identifier: NCT03461159) that was proposed to determine if operant conditioning can impact the efficacy of peripheral nerve stimulation on the upper extremities in both healthy and post-stroke patients.

## 3. The Appeal of Peripheral Nerve Stimulation

Peripheral nerve stimulation is an intriguing intervention because it represents a novel alternative to pharmaceutical interventions. Opioids are commonly utilized in the current pharmaceutical treatment of chronic pain and represent a major problem. Opioids are highly addictive and can be lethal if misused. It has been previously estimated that 16.5 million people worldwide use heroin or opium, with 23% of those users estimated to become dependent [19]. Historically, there has been a positive correlation found for a change in opioid-related deaths in a region with the percentage of people with opioid prescriptions in that same region [20]. Due to the overwhelming evidence supporting the dangers of opioids, it is best to seek alternative interventions, especially when it comes to the management of chronic conditions. Alternative interventions can include neuromodulation such as SCS and peripheral nerve stimulation. As compared to SCS, peripheral nerve stimulation is less invasive as leads do not need to be implanted in the epidural space [4]. In addition, peripheral nerve stimulation represents more options for the patient. Some patients may not want a neuromodulation device to be permanently implanted, and peripheral nerve stimulation presents the choice of a temporary system. Current clinical applications for peripheral nerve stimulation of the upper extremities include post-amputation pain, hemiparetic shoulder, CRPS, and neuropathy of the radial, median, and ulnar nerves.

## 4. Clinical Applications for Peripheral Nerve Stimulation in Upper Extremity Pain

### 4.1. Post Amputation Pain

Chronic pain after amputation is a very common complaint, reported in up to 90% of patients. Pain can be reported in the residual limb, phantom limb, or both. It is suggested that the primary mechanisms of pain in these patients are believed to involve supraspinal, spinal, and peripheral nerves [21]. Unfortunately, present treatments for post-amputation pain are largely pharmacologic and are rarely successful. Pharmacological treatments include N-Methyl-D-aspartate receptor antagonists (ketamine, dextromethorphan, memantine), anti-depressants (amitriptyline), opioids (morphine), Botulinum Toxin A injections, anticonvulsants (gabapentin), hormones (salmon calcitonin), and antihypertensives (nifedipine, propranolol, clonidine). Only opioids have been proven beneficial with adequate evidence. However as previously mentioned, they should not be used long-term as they come with the risk of dependence and/or tolerance. Other treatments consist of psychological options (e.g., hypnosis, cognitive behavior therapy [CBT], mirror visual feedback [MVF]), physical therapy (e.g., massage, tapping, acupuncture, ultrasound), and reconstructive surgery. These options either lack adequate evidence or are only feasible in select patient populations [22]. In 2014, Rauck et. al. published the use of peripheral nerve stimulation for lower extremity amputation pain [23]. Since then, multiple other clinicians have published similar studies with good results [24,25,26]. In these studies, the best results were seen in the use of high-frequency (10 kHz) stimulation. Implanted peripheral nerve cuff electrodes using the high-frequency stimulation resulted in a true conduction block. Additionally, there was blocking of action potentials from painful neuromas and pain without paresthesias. Although the majority of current studies are in the lower limb, it would be theorized that these techniques are translatable to the upper extremity amputation and there is a need for further research. Recently, Finneran et al. demonstrated a case where peripheral nerve stimulation of the brachial plexus significantly reduced post-amputation pain of the upper extremity that was sustained through the follow-up period of 180 days [27]. In 2015, a clinical trial (ClinicalTrials.gov Identifier: NCT02493842) was proposed in an attempt to study the effects of peripheral nerve stimulation on pain after upper extremity amputation (unilateral transradial amputation). However, no results have been posted and the status of the clinical trial is unknown.

### 4.2. Hemiparetic Shoulder Pain

Cerebrovascular accidents are the most common cause of hemiparesis and adult-onset disability. In a hemiparetic limb, shoulder pain is exceedingly common, with incidence rates ranging from 16% to 84% [28]. Current treatments can involve needling techniques (acupuncture and dry needling) or taping, although taping has been found to be largely ineffective [29,30]. Needling techniques have been found to be slightly more effective when applied directly to trigger points [31]. The etiology of shoulder pain in stroke is varied and often multifactorial. Rotator cuff injury, subluxation of the glenohumeral joint, CRPS, and brachial plexopathy are common and the varied and coexisting pathologies make it difficult to treat shoulder pain [28]. Due to the difficulty of treatment, hemiparetic shoulder is one of the most studied areas for peripheral nerve stimulator use.

The shoulder joint is primarily innervated by the suprascapular and axillary nerves, and the suprascapular nerve provides 70% of sensory supply to the posterior glenohumeral joint, capsule, and the overlying skin and motor innervation to the infraspinatus and supraspinatus muscles. The axillary nerve contributes to the sensory innervation of the shoulder joint and motor innervation of the deltoid and teres minor muscles [32]. These two nerves are the primary targets for current peripheral nerve stimulator research.

Both open surgical, fluoroscopic and ultrasound-guided lead placement techniques have been described. Open surgical lead placement involves dissecting down to the target nerve and placing the lead in close proximity to the suprascapular nerve. This technique provides for direct visualization of the target nerve and certainty of lead placement. However, an increased incidence of post-procedure pain and infection limits the utility of this approach as compared to the invasive ultrasound-guided technique, which is by far most commonly used and is discussed here.

The suprascapular nerve originates from the C5 and C6 spinal nerves and the upper trunk of the brachial plexus. Starting at its take-off from the upper trunk of the brachial plexus, the suprascapular nerve courses inferiorly beneath the omohyoid muscle, and turns posteriorly towards the suprascapular notch. In the suprascapular notch, the nerve often lies under the suprascapular ligament deep to the suprascapular artery, which is typically found above this ligament. From here, the nerve branches off and continues its course into the shoulder joint. Although the nerve could be targeted anywhere along this trajectory, it is most commonly targeted in the suprascapular notch due to the ease of finding anatomic landmarks, the nerve itself, and the placement of the leads [33].

For the ultrasound placement, either a linear or curvilinear transducer is used depending on the patient’s habitus. The transducer is placed in the coronal plane over the supraspinatus muscle and directed inferiorly to visualize the suprascapular notch with the suprascapular artery, ligament, and nerve. The needle is directed in-plane using a medial to lateral trajectory with care to avoid moving too far anteriorly to avoid pleural puncture. Once the needle is just past the nerve, a guide is placed and a dilator is used to open the space. The lead is placed adjacent to the nerve, ideally within 0.5 cm of the nerve with care taken to avoid placing it too close to the nerve to avoid motor stimulation [10,34].

The axillary nerve also originates from C5 and C6 spinal nerves and leaves the posterior cord of the brachial plexus. From there, it courses through the quadrangular space and wraps around the humerus at the inferior margin of the teres minor [35]. Similarly, the axillary nerve can be targeted anywhere along this trajectory, though is most frequently targeted at the posterior humerus. For ultrasound-guided lead placement of the axillary nerve, either a linear or curvilinear probe is used depending on the size of the patient. The probe is placed posteriorly over the humeral head and neck and a cross-section view of the infraspinatus can be seen cranially, while the teres minor is viewed caudally. At the inferior border of the teres minor, the axillary nerve and the circumflex artery are visualized. Doppler is used to identify vasculature. Both in-plane and out-of-plane approaches have been used to direct the needle adjacent to the nerve, employing a caudal to cranial, lateral to medial, or a medial to lateral approach. Ideally, the lead should be placed within 0.5 cm of the nerve with care to avoid placing it too close as this could cause motor stimulation [10,35].

Yu et al. first reported on the effectiveness of a fully implantable and rechargeable peripheral nerve stimulation system over 12-weeks in a patient with post-stroke pain using a microstimulator (Dakmed Peripheral Nerve Stimulator Model 750; Dakmed Inc., Buffalo, NY, USA) that was positioned near the axillary nerve in the quadrilateral space. This reduced shoulder pain from 8/10 before treatment to 4/10 after treatment and a further reduction to 3/10 at the 3-month follow-up. In addition, there was an improved passive range of motion and motor function [36]. This report sparked research into hemiparetic shoulder pain management with peripheral nerve stimulation. Subsequent case reports and case series have replicated the results of the above study reporting pain reduction ranging from 37% to 66% with peripheral nerve stimulation. One randomized control trial (RCT) with patients starting the trial with a mean pain score of 7.5/10, found that patients who underwent treatment with peripheral nerve stimulation had their pain scores reduced to an average of 3.0/10 at 16-weeks. For the patients that were undergoing usual care, their average pain score only reduced to 6.1/10 at 16-weeks [37]. In addition, patients that were undergoing peripheral nerve stimulation experienced significantly improved shoulder motor function as compared to the usual care treatment group. These results are supported by an additional RCT where patients in both groups (peripheral nerve stimulation and physical therapy) experienced shoulder motor function improvement, although the peripheral nerve stimulation group saw slightly greater improvements [38]. An exciting clinical trial (ClinicalTrials.gov Identifier: NCT02893267) is currently in progress with a randomized design with multiple arms: two treatment groups (peripheral nerve stimulation group and physical therapy group) and two sham groups (sham-peripheral nerve stimulation and sham-physical therapy). This high-powered RCT may provide significant evidence towards the efficacy of peripheral nerve stimulation for hemiparetic shoulder pain.

A recent clinical trial (ClinicalTrials.gov Identifier: NCT02928055) has also found a possible dose-dependent response to peripheral nerve stimulation for hemiparetic shoulder pain. This study found that the pain scores for the three groups immediately after the three-week treatment period were similar (9 h/day = 5.67; 6 h/day = 6; 3 h/day = 5.6). However, at the 3-month follow-up, the average pain score for participants in the 9 h/day peripheral nerve stimulation was 4.4/10 (*n* = 7), which was lower than the 6 h/day group’s average score of 5.2/10 (*n* = 7) and the 3 h/day groups’ average score of 6.5/10 (*n* = 5). Although this clinical trial provides some evidence of a dose-dependent response to peripheral nerve stimulation, future, more controlled studies are still warranted to strengthen the evidence.

### 4.3. Complex Regional Pain Syndrome (CRPS)

Formerly known as reflex sympathetic dystrophy (CRPS Type I) and causalgia (CRPS Type II), CRPS has an incidence rate of 26.2 per 100,000 person years [39]. This number rises significantly to approximately 7% in those who have injuries or surgery that is performed on limbs [40]. Despite, the relatively low overall incidence rate, CRPS is often a debilitating chronic pain disorder that devastates those who have it. The two types of CRPS differ in that Type I occurs with no known nerve damage whereas Type II does occur with nerve damage [41]. Accounting for around 1.2% of adult chronic pain patients, treatment interventions such as pharmacotherapy, sympathetic nerve blocks, or physical and psychological measures may not provide satisfactory pain relief [42]. In cases with intractable CRPS, peripheral nerve stimulation has been used with variable success. In a retrospective review of 165 patients with CRPS Type I or II, Chmiela et. al. reported a reduction in pain scores from a mean of 7.4 ± 1.6 to 5.5 ± 2.4 at 12-month follow-up. A total of 51% of patients reported functional improvement, and chronic opiate use amongst the cohort decreased from 62% at baseline to 41% with treatment [43]. Racz et al. described an open surgical approach for peripheral nerve stimulation implantation [44]. On-Point electrodes were placed adjacent to the median, ulnar, and radial nerves. The radial nerve was accessed via a dorsolateral approach in the spiral groove with incision over the triceps. Approximately a 4 cm segment of the radial nerve was dissected free. The electrode was attached to the perineurium with a fascial flap interposed between the electrode and the radial nerve from the medial head of the triceps. The median and ulnar nerves were accessed via an incision in the brachial groove. For each nerve about a 4 cm segment was dissected away from the adjacent tissue. Similarly, fascial flaps were taken from the adjacent tissue and the electrode-fascia complex was sutured to the perineurium. Reverberi et al. also utilized an open surgical approach for peripheral nerve stimulation implantation. Electrical nerve stimulation guided the surgical partial exposure of the nerve that was targeted and a 21 g needle was used. A total of six of the 15 patients in this case series were treated for CRPS Type II with the mean pain scores of the entire cohort reducing from 8.46/10 to 3.46/10. Specific nerves that were targeted included the brachial plexus (*n* = 2), ulnar nerve (*n* = 2), median nerve (*n* = 1), and radial nerve (*n* = 1) [45].

There are also case reports describing successful stimulation of the median, ulnar, and radial nerves using an ultrasound-guided percutaneous lead placement [46]. A case report from Narouze and Souzdalnitski utilized an ultrasound-guided and fluoroscopy-confirmed percutaneous peripheral nerve stimulation trial. The lead extended from the C6 to the T3 level to cover the cervical and upper thoracic sympathetic chain. The patient’s pain declined from a weekly average of 8/10 to 1/10 with significant functional gains. The lead placement was performed with the patient in a supine position with the neck extended. The ultrasound probe was placed transversely at the root of the neck to identify the C6 transverse process and relevant structures including the carotid artery, internal jugular vein, vertebral artery, and inferior thyroid artery. A 22 g blunt needle was then inserted in-plane to just anterior of the longus colli muscle. Then, using a guidewire and introducer, an octad percutaneous lead was placed to the final position parasagittally from C6-T3; fluoroscopy confirmed a satisfactory parasagittal lead position [47]. Bouche et al. also reported the use of an ultrasound-guided technique in the treatment of 16 patients with CRPS Type I or Type II. Leads were implanted near the supra-scapular nerve or the cervical nerve roots in the brachial plexus. The patients reported an average improvement of 68% in their pain scores [48]. More recently, Frederico and Frietas stimulated the brachial plexus in 10 patients with CRPS Type I or Type II. The mean pain scores reduced from 8.9/10 to 3.8/10 at the one year follow-up [49].

### 4.4. Radial, Median, and Ulnar Neuropathy

Theoretically, these individual nerves can be targeted to treat chronic pain secondary to previous nerve insult to the area, as well as nerve entrapment in which more extensive surgery is either not feasible, or if the patient continues to report pain after surgical correction. A prospective study on peripheral nerve stimulation of the median nerve for the treatment of carpal tunnel syndrome targeting the median nerve near the wrist enrolled eight patients. A total of two of the eight patients reported significant longstanding relief of their carpal tunnel symptoms following peripheral nerve stimulation implantation [50]. Numerous clinical trials (e.g., ClinicalTrials.gov Identifier: NCT04246216), many of which include randomization and multiple arms, have been registered to study peripheral nerve stimulation for carpal tunnel syndrome, so there is hope for more definitive evidence in the future. Singh et al. also utilized peripheral nerve stimulation that was targeted at the median nerve for the treatment of upper extremity pain secondary to human papillomavirus papillomatosis in a case report. The patient reported an 80% improvement in pain and significant improvement in function overall [51]. Another study aimed to look at patients with painful radial neuropathy with lateral epicondylalgia. This study showed promising results for patients with unilateral unremitting lateral elbow pain with decreased pain and improved function [52]. There are case reports on peripheral nerve stimulation for ulnar neuropathy in patients with unremitting pain from cubital tunnel syndrome [53]. Singh et al. demonstrated a similar case of carpal tunnel and cubital tunnel syndrome where peripheral nerve stimulation of the median and ulnar nerves improved the patient’s pain by 50% at one year follow-up [51]. Peripheral stimulation of the ulnar nerve may be a good strategy to obtain pain relief after other options have been exhausted including surgical release and pharmacologic treatments. A clinical trial (ClinicalTrials.gov Identifier: NCT02566616) was proposed to further study peripheral stimulation of the ulnar nerve to treat cubital tunnel syndrome, however, no participants were enrolled and the proposal was withdrawn.

The technical aspect of placing devices is often relatively straightforward, so long as the target nerve can be adequately identified on ultrasound. These nerves are targeted proximal to the site of their entrapment or irritation, and the neurostimulator leads are guided in proximity to the nerve under direct ultrasound visualization. Some peripheral nerve stimulator devices can be placed via anatomic landmarks that are identified on fluoroscopy, however, due to the smaller diameter of these nerves in the peripheral upper extremity, ultrasound is the imaging modality of choice when placing the leads for precision [51].

## 5. Complications of Peripheral Nerve Stimulation

Historically, complication rates of peripheral nerve stimulators have been rather high, although the morbidity that is associated with these complications is typically low. The high complication rate is largely attributed to the use of SCS that is adapted for use in the periphery, as SCS leads were not developed to take the abuse that is placed on a lead in the periphery as compared to the epidural space [54]. Today, with newer devices on the market, complication rates seem to be lower, though data are very limited. Generally, peripheral nerve stimulators are considered safe and effective, despite a lack of sufficient data [55].

Complications of peripheral nerve stimulators can be largely classified into two categories: hardware-related or biological in origin. Hardware complications are primarily lead-related such as lead migration, lead breakage, or lead disconnection. Lead migration rates ranged from 2% to 13% in a retrospective analysis when leads were sutured to the deep fascia, with 2.1% necessitating surgical revision. It should be noted that these percentages were in all locations of peripheral nerve stimulator placement, not just in the upper extremity. Lead migration was most prevalent in stimulators in the head and neck [54]. Choi et. al. published a case report of lead fracture as it crossed the shoulder joint [55,56]. Rarely reported are complications with the pulse generator including battery malfunction or positioning issues/pain. A 13 year retrospective case series by Warner et. al. looking specifically for complications that were associated with peripheral nerve stimulators documented that 20/72 patients required device explantation for various reasons, with the most common being infection (*n* = 5). There were 17 patients who required lead revision, with lead migration being the cited reason in nine cases, followed by device malfunction (*n* = 5), lead/anchor erosion (*n* = 5), and infection (*n* = 4) [57]. However, this study was performed using SCS leads rather than newer, current devices that are designed for peripheral nerve stimulation.

Biological complications include infection, hematoma or seroma formation, pain, or nerve damage. Pain is by far the most frequently cited complication. Pain that is related to the internal pulse generator has been reported as a complication in up to 25% of cases, though the use of external pulse generators has largely eliminated this problem. Infections, both superficial and deep, have been reported with rates ranging from 1–6% of cases. Of these, many were able to be treated with oral antibiotic therapy alone although explantation was required in some individuals [54]. Issues that were related to skin erosion have been cited as well in up to 7% of cases, though these were seemingly related more to internal pulse generators again. There were no reports of neurologic damage with the use of peripheral nerve stimulation. A summary of all clinical implications and complications of peripheral nerve stimulation can be found in Table 1.

## 6. Conclusions

This is an exciting time for peripheral nerve stimulation. The availability of ultrasound to visualize soft tissues and peripheral nerves has made it possible to safely and accurately position leads in proximity to the target nerve/s using minimally invasive techniques. Advances in technology and the development of flexible fine peripheral nerve stimulator leads and neurostimulation technologies allow for safe percutaneous lead placement. As a result, new avenues and treatment options for a variety of painful conditions with the potential for improved outcomes are now available to pain physicians.

## Figures and Tables

**Table 1:** Summary of clinical applications and complications.

Clinical Applications of Peripheral Nerve Stimulation	
Post-Amputation Pain	- Reported in up to 90% of amputation patients [21] - Present treatments are mostly pharmaceutical and ineffective [22] - Supraspinal, spinal, and peripheral nerves are believed to be involved [21] - Current research for peripheral nerve stimulation has been focused on lower extremity pain o Reductions in pain have been reported in current lower extremity pain studies [23,24,25,26] o Techniques for lower extremities may be translatable for application to the upper extremities [27]
Hemiparetic Shoulder Pain	- Typically caused by cerebrovascular accidents [28] - Difficult to treat due to varied and multifactorial etiology [28] - One of the most studied areas of peripheral nerve stimulation [28] - Suprascapular and axillary nerves are the major nerves studied [32] - Ultrasound-guided is the most commonly used implantation technique [10,34] - Current research for peripheral nerve stimulation has found reductions in pain o Including two completed RCTs [37,38] o Another RCT is in progress (ClinicalTrials.gov Identifier: NCT02893267) - Current research for peripheral nerve stimulation also found improvements in motor function [36,37,38] - Possible dose-dependent response (ClinicalTrials.gov Identifier: NCT02928055)
CRPS	- Present in 7% of those with injuries or surgery performed on limbs [40] - 1.2% of adult chronic pain patients [42] - Current treatments may not provide adequate pain relief [42] - Radial, median, and ulnar nerves are involved [46] - Current research for peripheral nerve stimulation has found variable success o Recent studies have shown peripheral nerve stimulation to be largely successful in reducing pain [43,44,45,46,47,48,49]
Radial, Median, and Ulnar Neuropathy	- Current research for peripheral nerve stimulation has found success for reductions in pain and improvements in motor function o Peripheral nerve stimulation targeting the median nerve is widely studied with multiple clinical trial in progress [50,51] o No clinical trials were found for peripheral nerve stimulation targeting the radial or ulnar nerves - Stimulation of the ulnar nerve may be a good strategy for pain relief if other interventions have been exhausted [51,53] - Ultrasound-guided is the most precise implantation technique [51]
Peripheral Nerve Stimulation Complications	
Hardware-related	- Hardware-related complications include lead migration, lead fractures, and lead disconnection [54] - For peripheral nerve stimulation implantation at all locations (not just upper extremity), lead migration rates are 2% to 13% [54,57] - Battery malfunction and positioning issues/pain is rarely reported [55]
Biological	- Biological complications consist of infection, hematoma or seroma formation, pain, or nerve damage [54] - Infection is the most common reason for device explanation o Infections are reported in 1% to 6% of cases [54] - Skin erosion-related issues are reported in 7% of cases (although this mostly applied to patients with an internal pulse generator) [54] - Pain is the most common biological complication, cited in up to 25% of peripheral nerve stimulation patients with an internal pulse generator [54] - No reports of neurological damage due to peripheral nerve stimulation [54] - Implementation of an external pulse generator has greatly reduced many of these complications [55]

## Data Availability

Data are available upon request to the corresponding author.
